# Protocol for a phase II study to evaluate the efficacy and safety of nivolumab as a postoperative adjuvant therapy for patients with esophageal cancer treated with preoperative docetaxel, cisplatin plus 5-fluorouracil treatment (PENTAGON trial)

**DOI:** 10.1371/journal.pone.0299742

**Published:** 2024-04-18

**Authors:** Hironobu Goto, Taro Oshikiri, Takashi Kato, Yoshiaki Nagatani, Yohei Funakoshi, Yasufumi Koterazawa, Ryuichiro Sawada, Hitoshi Harada, Naoki Urakawa, Hiroshi Hasegawa, Shingo Kanaji, Kimihiro Yamashita, Takeru Matsuda, Hironobu Minami, Yoshihiro Kakeji

**Affiliations:** 1 Division of Gastrointestinal Surgery, Department of Surgery, Graduate School of Medicine, Kobe University, Kobe, Hyogo, Japan; 2 Division of Medical Oncology and Hematology, Department of Medicine, Graduate School of Medicine, Kobe University, Kobe, Hyogo, Japan; Maria Sklodowska-Curie National Research Institute of Oncology Krakow, POLAND

## Abstract

**Background:**

In Japan, preoperative adjuvant chemotherapy followed by surgical resection is the standard treatment for patients with locally advanced esophageal squamous cell carcinoma. However, the risk of recurrence after surgical resection remains high. Although a randomized controlled trial evaluating the efficacy of nivolumab, a fully human monoclonal anti-programmed death 1 antibody, as postoperative adjuvant therapy after neoadjuvant chemoradiotherapy and surgery established its superior efficacy as adjuvant therapy, the efficacy for patients who received preoperative adjuvant chemotherapy has not been demonstrated. This study aims to elucidate the efficacy and safety of nivolumab as postoperative adjuvant therapy for patients with esophageal squamous cell carcinoma after preoperative adjuvant chemotherapy with docetaxel and cisplatin plus 5-fluorouracil followed by surgical resection.

**Methods:**

This study is a multi-institutional, single-arm, Phase II trial. We plan to recruit 130 esophageal squamous cell carcinoma patients, who have undergone preoperative adjuvant chemotherapy with docetaxel and cisplatin plus 5-fluorouracil followed by surgical resection. If the patient did not have a pathological complete response, nivolumab is started as a postoperative adjuvant therapy within 4–16 weeks after surgery. The nivolumab dose is 480 mg/day every four weeks. Nivolumab is administered for up to 12 months. The primary endpoint is disease-free survival; the secondary endpoints are overall survival, distant metastasis-free survival, and incidence of adverse events.

**Discussion:**

To our knowledge this study is the first trial establishing the efficacy of nivolumab as postoperative adjuvant therapy for patients with esophageal squamous cell carcinoma after preoperative adjuvant chemotherapy with docetaxel and cisplatin plus 5-fluorouracil followed by surgical resection. In Japan, preoperative adjuvant chemotherapy followed by surgery is a well-established standard treatment for resectable, locally advanced esophageal squamous cell carcinoma. Therefore, developing an effective postoperative adjuvant therapy has been essential for improving oncological outcomes.

## Introduction

Esophageal cancer is one of the most common malignancies worldwide. To improve survival outcomes, multidisciplinary treatments have been developed [1, 2]. In Japan, the effect of preoperative adjuvant chemotherapy for locally advanced esophageal squamous cell carcinoma (ESCC) was evaluated in a randomized controlled trial (JCOG9907), which compared survival outcomes following postoperative versus preoperative adjuvant chemotherapy with cisplatin and 5-fluorouracil (FP) for locally advanced ESCC [3]. The superior efficacy of preoperative chemotherapy has been established. In a subsequent randomized controlled trial (JCOG1109), a survival benefit was demonstrated in patients receiving preoperative docetaxel and cisplatin plus 5-fluorouracil (DCF) relative to FP therapy [4]. Based on these results, preoperative adjuvant chemotherapy with DCF followed by surgical resection has been the standard treatment for patients with locally advanced ESCC.

In contrast, in Western countries, the survival benefits of neoadjuvant chemoradiotherapy (NACRT) over surgery alone have been demonstrated in several clinical trials, and NACRT is the standard treatment for patients with esophageal cancer [5–7]. However, the risk of recurrence after NACRT followed by surgery remains high, especially in patients who do not have a pathological complete response (pCR) [8]. Therefore, a randomized controlled phase III trial that evaluated the efficacy of nivolumab, a fully human monoclonal anti-programmed death 1 antibody, as postoperative adjuvant therapy after NACRT and surgery for esophageal cancer was designed. Nivolumab adjuvant therapy showed superior efficacy [9].

However, the efficacy of nivolumab in patients who received preoperative adjuvant chemotherapy for esophageal cancer has not been demonstrated, and a previous randomized controlled trial only assessed its efficacy in patients who underwent NACRT. In Japan, a survival benefit was not established in patients receiving NACRT compared to preoperative FP therapy in the JCOG1109 trial [4]. Therefore, the standard treatment for patients with locally advanced ESCC is preoperative adjuvant chemotherapy followed by surgical resection. Thus, establishing the efficacy of nivolumab as postoperative adjuvant therapy for patients with ESCC after preoperative adjuvant chemotherapy with DCF followed by surgical resection is necessary.

## Materials and methods

### Objectives

This study aimed to elucidate the efficacy and safety of nivolumab as a postoperative adjuvant therapy for patients with ESCC after preoperative adjuvant chemotherapy with DCF followed by surgical resection.

### Study design

This study is a multi-institutional (nine specialized centers), single-arm, Phase II trial. This multi-institutional trial protocol was approved by the Institutional Review Board and Ethics Committee of Kobe University in December 2022 and by the institutional review board of each participating institution before initiating patient recruitment. The principal investigator will manage the data of patients enrolled from nine hospitals using the Electronic Data Capture system. The trial was registered in the Japan Registry of Clinical Trials under jRCT1051220153. Monitoring is conducted to evaluate the study’s progress, improve data integrity, and record patient safety. The monitoring officers designated by the principal investigator conduct data management, central monitoring, and statistical analyses. The officers received educational training on the Ethical Guidelines for Medical and Health Research and fully understood the content of this study.

### Study population

The staging of ESCC was based on the seventh edition of the Union for International Cancer Control tumor node metastasis cancer staging system [10]. The inclusion and exclusion criteria are presented below in Table 1.

**Table 1:** Inclusion and exclusion criteria.

Inclusion criteria	Primary lesions are located in the thoracic esophagus and histologically proven squamous cell carcinoma.
	Clinical stage is 2 or 3 (based on esophagogastroduodenoscopy, computed tomography, and positron emission tomography).
	Preoperative adjuvant chemotherapy with DCF is administered, followed by complete surgical resection, and residual pathological disease with a tumor and node classification, at least in the resected specimens, is observed.
	Age is over 18 years at the registration date.
	An Eastern Cooperative Oncology Group performance status of 0 or 1.
	Within 4–16 weeks following surgery.
	Disease-free status is confirmed within four weeks of registration (observed based on chest and abdominal computed tomography examinations).
	Sufficient organ functions confirmed within 14 days of registration. (i) Absolute neutrophil count ≥ 1500/mm3 (ii) Platelets ≥ 10.0×104/mm3 (iii) Hemoglobin ≥ 9.0 g/dL (iv) Creatinine: No higher than the upper limit of normal×1.5, or creatinine clearance > 50 mL/min (Cockcroft-Gault equation) (v) Aspartate aminotransferase/alanine aminotransferase: No higher than the upper limit of normal×3.0 (vi) Total bilirubin: No higher than the upper limit of normal×1.5
	Written informed consent.
Exclusion criteria	Infectious disease requiring systemic treatment.
	Suffering from an active autoimmune disease.
	Requirement for systemic steroid medication (≥ 10 mg/day of prednisolone) or immunosuppressants.
	Currently or previously receiving treatment with immune checkpoint inhibitors.
	Severe interstitial lung pneumonia or lung fibrosis.
	For females, those unwilling to use contraception during the treatment period and expecting and breastfeeding mothers.
	Patients who are not candidates for surgery for any reason.

### Study protocol

**Fig 1:**
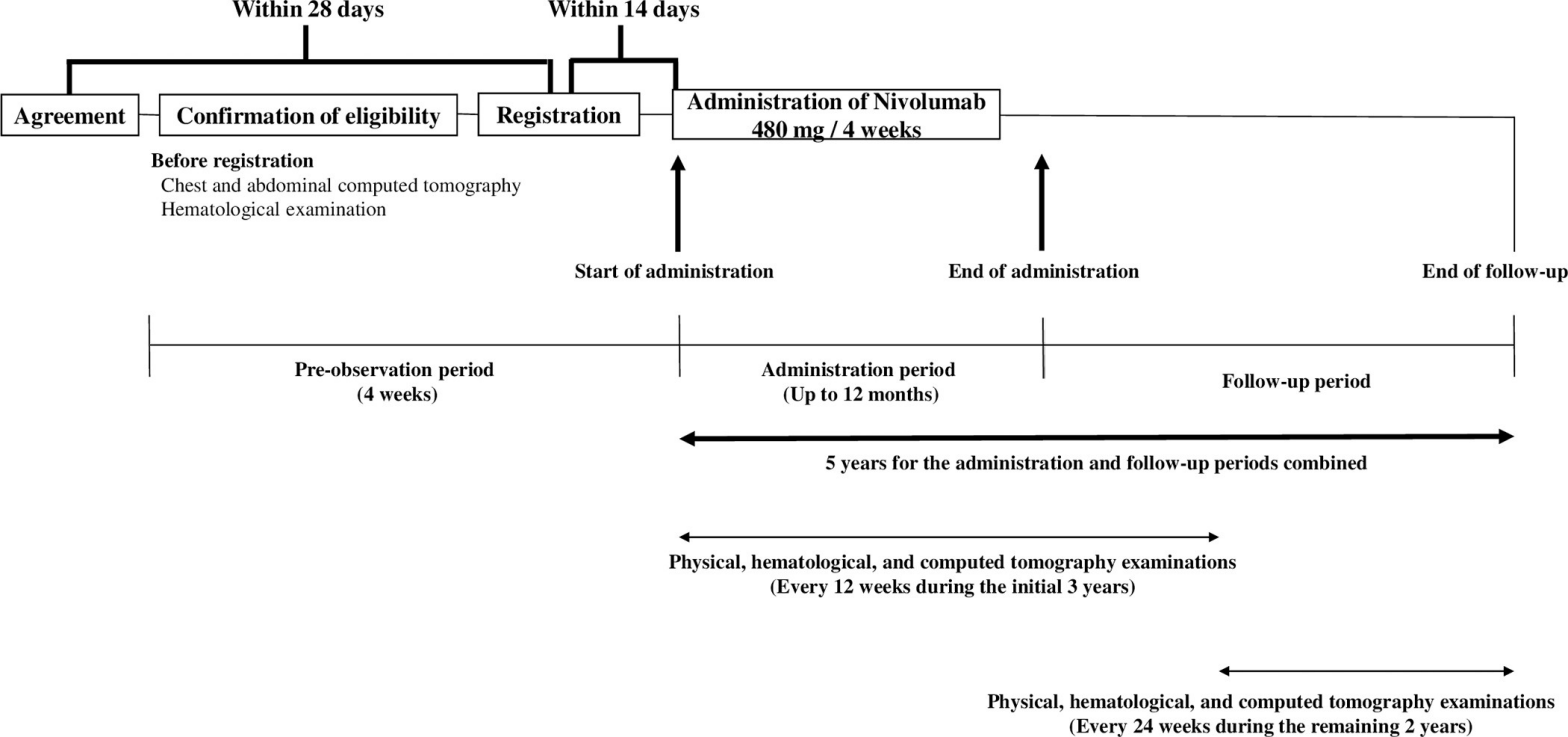
The time schedule of enrollment, administration of nivolumab, and follow-up for the PENTAGON trial.

All patients receive at least one course of preoperative DCF therapy. This study does not specify the number of courses and drug doses related to preoperative DCF therapy. After preoperative DCF therapy, subtotal thoracic esophagectomy and regional lymphadenectomy with right thoracotomy are performed. Thoracoscopic and robot-assisted esophagectomies are acceptable, but the transhiatal and mediastinoscopic approaches are not allowed. The regional lymph nodes include the thoracic, cervical (paraesophageal, paratracheal, subcarinal, and mediastinal), and perigastric lymph nodes. After R0 resection, if the patient did not have a pCR, nivolumab is started as a postoperative adjuvant therapy within 4–16 weeks after surgery. The time schedule is shown in Fig 1. The nivolumab dose is 480 mg/day every four weeks. Nivolumab is administered for up to 12 months unless interrupted by unacceptable toxicity, unequivocal relapse, or withdrawal of the patient’s consent. Hematological examinations are performed before the initiation of nivolumab administration. If sufficient organ function is not confirmed by hematological examination (i-vi), nivolumab administration is skipped. Dose reduction or modification of nivolumab is not recommended. The details of the treatment discontinuation and interruption criteria are shown in Table 2.

**Table 2:** The treatment discontinuation and interruption criteria for nivolumab using National Cancer Institute Terminology Criteria for Adverse Events version 5.0.

		Interruption	Resuming administration	Discontinuation
Interstitial pneumonia		Grade 1	Recovery to grade 0	Grade 2 or higher
Colitis / Diarrhea		Grade 2	Recovery to grade 1 or below	Grade 3 or higher
Liver dysfunction	AST/ALT	Grade 2	Recovery to grade 1 or below	Grade 3 or higher
	Total-bilirubin	Grade 2	Recovery to grade 1 or below	Grade 3 or higher
Thyroid dysfunction	Asymptomatic	Continue to administration. Consider starting hormone replacement therapy.		
	
	Symptomatic	Discontinue administration and start hormone replacement therapy. If the patients have recovered to grade 1 or below, consider resuming administration.		
	
Pituitary / adrenal dysfunction	Asymptomatic	Continue to administration. Consider starting hormone replacement therapy.		
	
	Symptomatic	Discontinue administration and start hormone replacement therapy. If the patients have recovered to grade 1 or below, consider resuming administration.		
	
Type 1 diabetes mellitus		Discontinue administration and start insulin replacement therapy. If the blood glucose levels has recovered to baseline, consider resuming administration.		
	
Kidney dysfunction	Creatinine	Grade 2 or 3	Recovery to grade 1 or below	Grade 4
Neuropathy		Grade 2	Recovery to below baseline grade	Grade 3 or higher
Dermopathy		Grade 3	Recovery to grade 1 or below	-
Myocarditis / Myositis		Grade 2	Grade 0	Grade 3 or higher
Cerebritis		-	-	Grade 3 or higher

*AST*: Aspartate aminotransferase; *ALT*: Alanine aminotransferase

Adverse events are evaluated throughout the treatment period using the National Cancer Institute Terminology Criteria for Adverse Events version 5.0 (CTCAE v 5.0). The treatment protocol is discontinued if nivolumab administration cannot be resumed within 42 days of the last administration. There is no additional treatment for ESCC after the completion of postoperative adjuvant therapy unless there is a confirmed relapse. Immunosuppressive drugs, systemic steroid medication (≥ 10 mg/day of prednisolone), and other antitumor therapies are prohibited during nivolumab administration.

### Endpoints

The primary endpoint is disease-free survival (DFS). DFS is defined as the period from registration to relapse, second cancer, or death from any cause. The secondary endpoints are overall survival (OS), distant metastasis-free survival (DMFS), and incidence of adverse events. OS is defined as the period from registration to death due to any cause. DMFS is defined as the period from the registration date to distant metastasis. The incidence of adverse events is determined using CTCAE v 5.0. The frequency of worst-grade adverse events during postoperative adjuvant therapy is also calculated. For the exploratory endpoint, the relationship between programmed cell death ligand 1 expression categorized by tumor proportion score and OS is evaluated.

### Follow-up

All patients included in the study are followed up for five years. Physical and hematological examinations are conducted every 12 weeks during the initial three years and every 24 weeks in the remaining two years. Enhanced chest and abdominal computed tomography examinations are performed at the same intervals.

### Statistical analysis

This phase II study investigates the safety and oncologic outcomes of nivolumab as a postoperative adjuvant therapy for patients with ESCC after preoperative adjuvant chemotherapy with DCF followed by surgical resection. The three-year DFS in the preoperative adjuvant DCF therapy arm excluding pCR cases in the JCOG1109 trial was 64% [4]. Assuming this value as a historical control, we would expect a 10% higher three-year DFS with nivolumab as postoperative adjuvant therapy. In this Phase II trial, the sample size for the analysis is calculated as 123 cases with a power of 80%, a one-sided alpha level of 5%, a planned recruitment period of three years, and a follow-up period of five years. Finally, the sample size was set at 130 cases, assuming a dropout rate of 10% and some ineligible cases. The Kaplan—Meier method assesses DFS, OS, and DMFS. The safety endpoint is the frequency of disease occurrence, tabulation tables are prepared for the endpoints, and exact two-sided 95% confidence intervals for binomial distribution are calculated for percentage estimation.

## Interim analysis

An interim analysis is not performed in this study.

### Independent Data Monitoring Committee

No Independent Data Monitoring Committee is established for this study.

### Protection of personal information

In this study, the subject identification code list will be used to link the database and clinical research documents to the original data of the research subjects. The principal investigator will keep the subject identification code list in a lockable location and separate from other clinical research documents. Limited subject information, such as gender and date of birth, may be used to identify research subjects and to verify the accuracy of the subject identification code list within the limits of all applicable laws and regulations. The principal investigator will not provide the list of research subject identification codes outside the respective institution.

### Ethics approval and consent to participate

Oral and written informed consent will be obtained from every participant by the treating doctor. The study protocol was approved by the Institutional Review Board and Ethics Committee of Kobe University on 22 December 2022. The study will be performed in accordance with the Declaration of Helsinki [11]. This trial was registered in the Japan Registry of Clinical Trials with identification number jRCT1051220153 on 22 January 2023.

## Discussion

In Japan, preoperative adjuvant chemotherapy followed by surgery is a well-established standard treatment for resectable, locally advanced ESCC [3, 4]. Therefore, developing an effective postoperative adjuvant therapy has been essential for improving oncological outcomes. To our knowledge, this study is the first trial establishing the efficacy of nivolumab as postoperative adjuvant therapy for patients with ESCC after preoperative adjuvant chemotherapy with DCF followed by surgical resection. In the CheckMate 577 trial, nivolumab was administered at a dose of 240 mg every 2 weeks for 16 weeks, followed by 480 mg every 4 weeks beginning at week 17 [9]. We consider the safety and efficacy of nivolumab administration at doses of 240 mg every 2 weeks and 480 mg every 4 weeks to be equivalent, so we standardized them. In Western countries, NACRT is the standard treatment for patients with esophageal cancer [5–7]. Therefore, a comparison of the efficacy of preoperative adjuvant chemotherapy and chemoradiotherapy is very important. Because preoperative adjuvant chemotherapy is the standard treatment for patients with esophageal cancer in Japan, NACRT cases are excluded from this study [4].

In the JCOG1109 trial, three courses of preoperative DCF therapy were administered, and the precise drug doses were specified [4]. However, this study did not provide specific details regarding the number of courses and drug doses for preoperative DCF therapy. Because DCF therapy is expected to have high toxicity and low treatment completion rates, conducting the study under real-world clinical conditions would be more appropriate without prescribing the number of courses or drug doses. In the CheckMate 577 trial, as in this study, pCR cases were excluded [9]. This trial population consisted of patients with residual pathological disease and a high risk of recurrence, which is reported in 70–75% of patients who do not have a pathological complete response after NACRT and surgery [1]. The PENTAGON trial was designed with the same concept, but it included preoperative adjuvant DCF therapy instead of NACRT and used the preoperative adjuvant DCF therapy arm excluding pCR cases in JCOG1109 as the historical control. We consider that this approach is justified.

This study is a multi-institutional, single-arm, Phase II trial with no control or chemotherapy- arm. Despite several retrospective studies suggesting a clinical benefit with adjuvant chemotherapy after induction therapy followed by surgical resection, the routine use of postoperative adjuvant chemotherapy has not been recommended [12, 13]. Prospective data are lacking to support the use of adjuvant chemotherapy. Postoperative adjuvant chemotherapy is often challenging to administer safely in patients who have already received extensive treatment, including toxic chemotherapy followed by esophagectomy, which may require a recovery period of a few months. On the other hand, postoperative adjuvant immunotherapy was shown to be safe and maintain quality of life [9].

## Conclusions

We designed this phase II trial to evaluate the efficacy and safety of nivolumab as a postoperative adjuvant therapy for patients with ESCC treated with preoperative DCF therapy.

## Supporting information

S1 Checklist Recommended items to address in a clinical trial protocol and related documents. (DOC)

S1 Protocol Full protocol of the PENTAGON trial. (DOCX)

S1 Fig The time schedule of enrolment, administration of nivolumab, and assessments for the PENTAGON trial. (DOC)
