# Supplementary material for: Protocol for a phase II study to evaluate the efficacy and safety of nivolumab as a postoperative adjuvant therapy for patients with esophageal cancer treated with preoperative docetaxel, cisplatin plus 5-fluorouracil treatment (PENTAGON trial)
Source: PLoS One. 2024 Apr 18;19(4):e0299742. doi: 10.1371/journal.pone.0299742 (PMC11025784; doi:10.1371/journal.pone.0299742)
Supplement: S1 Protocol — (DOCX) [file pone.0299742.s002.docx]

**Research plan**

**Phase II Study to Evaluate the Efficacy and Safety of Nivolumab as Postoperative Adjuvant Therapy in Patients with Esophageal Cancer Treated with Preoperative DCF Treatment**

**PENTAGON trial**

Version 2.1 December 22, 2022

| **Principal investigator** |
| --- |
| Taro Oshikiri  Division of Gastrointestinal Surgery, Department of Surgery, Kobe University  7-5-2, Kusunoki-cho, Chuo-ku, Kobe, Hyogo, 650-0017, Japan |
| **Research bureau** |
| Taro Oshikiri  7-5-2, Kusunoki-cho, Chuo-ku, Kobe, Hyogo, 650-0017, Japan  Yoshiaki Nagatani  7-5-2, Kusunoki-cho, Chuo-ku, Kobe, Hyogo, 650-0017, Japan |

## Overview of clinical research

| **Title** | Phase II Study to Evaluate the Efficacy and Safety of Nivolumab as Postoperative Adjuvant Therapy in Esophageal Cancer Patients Treated with Preoperative DCF Therapy |
| --- | --- |
| **Object** | To evaluate the efficacy and safety of postoperative adjuvant therapy with nivolumab in patients with esophageal cancer after preoperative chemotherapy and complete resection. |
| Research design | This is a single-arm, open-label, multicenter Phase II study. |
| Subject | Inclusion criteria： 1. The primary lesion is located in the thoracic esophagus and is histologically diagnosed as squamous cell carcinoma by endoscopic biopsy.  2. Complete resection was performed after preoperative DCF (docetaxel, cisplatin, 5-FU) therapy, but no pathologic complete response was achieved.  3. The applicant is at least 18 years of age on the date of registration.  4. Gender is not required.  5. Performance status is 0 or 1 by ECOG criteria.  6. Patients can be enrolled between 4-16 weeks (28-112 days postoperatively) after complete resection.  7. Patients are confirmed disease-free by thoracoabdominal CT within 4 weeks prior to enrollment.  8. The most recent laboratory values within 14 days prior to registration (the same day of the week two weeks prior to the registration date is acceptable) meet all of the following  ・ Absolute neutrophil count ≧ 1,500/mm^3^  ・ Platelets ≧ 10.0×10^4^ /mm^3^  ・ Hemoglobin ≧ 9.0 g/dL  ・ Creatinine：No higher than the upper limit of normal × 1.5, or creatinine clearance ＞ 50mL/min (Cockcroft-Gault equation)  ・ AST/ALT：No higher than the upper limit of normal × 3.0  ・ Total bilirubin：No higher than the upper limit of normal × 1.5  9. The patient's free and voluntary written consent to participate in this clinical research has been obtained. Exclusion criteria： 1. Infectious disease requiring systemic treatment.  2. Suffering from an active autoimmune disease.  3. Requirement for systemic steroid medication（≧ 10 mg/day of prednisolone）or immunosuppressants.  4. Currently or previously receiving treatment with immune checkpoint inhibitors.  5. Severe interstitial lung pneumonia or lung fibrosis.  6. The patient is deemed inappropriate by the person in charge of this clinical research.  7. Unwilling to use contraception and expecting and breastfeeding mothers. |
| Primary endpoint | Disease-free-survival (DFS) |
| Secondary endpoints | **【Efficacy endpoints】**  ・Overall survival (OS)  ・Distant metastasis-free survival (DMFS)  **【Safety endpoint】**  ・Incidence of adverse events |
| **Protocol treatment** | The dosage regimen is 480 mg of nivolumab administered intravenously at 4-week intervals. The duration of administration should be up to 12 months. |
| **Number of cases** | 130 cases |
| **Clinical research period** | From the date of publication of the implementation plan to July 31, 2031  Registration period: Date of publication of implementation plan～July 31, 2025  Follow-up period: 5 years from the last registration date |
| **Contact information** | **Research bureau**  Taro Oshikiri  7-5-2, Kusunoki-cho, Chuo-ku, Kobe, Hyogo, 650-0017, Japan  Yoshiaki Nagatani  7-5-2, Kusunoki-cho, Chuo-ku, Kobe, Hyogo, 650-0017, Japan |

## Abbreviation

| **Abbreviation** | **English** |
| --- | --- |
| TPS | Tumor Proportion Score |
| DFS | Disease-Free Survival |
| DMFS | Distant Metastasis-Free Survival |
| OS | Overall Survival |
| PD-L1 | Programmed cell Death Ligand-1 |
| jRCT | Japan Registry of Clinical Trials |

# 2．Background and Objectives

## 2.1　Background

Thoracic esophageal cancer in Japan is a malignant disease that predominantly affects men in their 60s to 70s, with an annual incidence of approximately 28,500 cases in Japan. The results of JCOG9907, "Randomized Comparison of Preoperative Adjuvant Cisplatin + 5-FU (CF) and Postoperative Adjuvant Chemotherapy for Stage II and III Thoracic Esophageal Cancer," showed that preoperative adjuvant chemotherapy plus surgery was the standard treatment. However, the 5-year overall survival rate was 55%, which was not good. Subsequently, JCOG1109, "Phase III Comparison of Preoperative CF/Preoperative DCF/Preoperative CF-RT for Clinical Stage IB/II/III (excluding T4) Thoracic Esophageal Cancer (UICC 7th)," demonstrated a favorable 3-year overall survival rate (72%) in the preoperative DCF plus surgery group, and DCF therapy is now the standard of care for preoperative treatment. On the other hand, preoperative chemoradiotherapy plus surgery is the standard of care in Europe and the United States, and a "global phase III study of the efficacy of nivolumab in esophageal cancer or esophagogastric junction cancer after preoperative chemoradiotherapy and complete resection" was conducted in that population, showing the efficacy of nivolumab versus placebo as postoperative adjuvant therapy. Median DFS; 22.4 vs. 11.0 months, hazard ratio 0.69 (95% confidence interval 0.56-0.86), P=0.0003]. Accordingly, the use of nivolumab as adjuvant therapy for esophageal cancer patients who have failed to achieve pathologic complete response was approved by the Japanese government in November 2021. However, considering that the preoperative treatment in this study was chemoradiotherapy, the efficacy of nivolumab in patients who received preoperative chemotherapy has not been determined, and that chemotherapy is the standard of preoperative treatment in Japan, the efficacy of postoperative nivolumab after preoperative chemotherapy in this setting needs to be determined the study was designed to investigate the efficacy of nivolumab after preoperative chemotherapy. Therefore, we will evaluate the efficacy in an exploratory manner in this study.

## 2.2　Objectives

This clinical study will evaluate the safety and efficacy of nivolumab as an exploratory adjuvant therapy to improve the prognosis of patients with resected esophageal cancer who have failed to achieve a pathologic complete response to preoperative chemotherapy.

### 2.2.1　Primary endpoint

Evaluate disease-free-survival (DFS) in postoperative adjuvant therapy with nivolumab.

### 2.2.2　Secondary endpoints

・Evaluate overall survival (OS).

・Evaluate distant metastasis-free survival (DMFS).

・Evaluate incidence of adverse events.

### 2.2.3　Purpose of Exploration

- PD-L1（TPS）, CD103^+^CD8^+^Tcell, Foxp3^+^CD4^+^Tcell, Bcl6^-^CD4^+^Tcell

# 3．Summary of Test Drugs

　For drug information, please refer to the latest drug package inserts at the following URL.

http://www.pmda.go.jp/PmdaSearch/iyakuSearch/

## 3.1 Study drug information

**1) Name of the drug**

Fully human monoclonal anti-programmed death 1 antibody: Nivolumab (Opdivo)

（Manufactured and marketed by Ono Pharmaceutical Co.）

**2) Route of administration, dosage and administration period**

Nivolumab is administered at a dose of 480 mg intravenously at 4-week intervals. The duration of administration should be up to 12 months.

**3) Target population (age group, gender, disease, etc.)**

Postoperative esophageal cancer patient who failed to achieve pathologic complete response to preoperative adjuvant therapy.

**4) Clinically significant findings on the efficacy and safety of the drug, etc., obtained from non-clinical studies, other clinical studies, etc.**

In a global phase III study of nivolumab in esophageal cancer or esophagogastric junction cancer after preoperative adjuvant chemoradiation and complete resection, nivolumab significantly prolonged prognosis compared with placebo (median disease-free survival: 22.41 months in the nivolumab group versus 11.04 months in the placebo group; HR: 0.69, 95% CI 0.56-0.86). On the other hand, no deaths were proven to be causally related to nivolumab, although Grade 3-4 adverse events were observed in 13.3% of patients.

**5) Known and potential benefits and disadvantages of the drug, etc. administered**

**Expected benefits**

The drugs to be used in this study are approved and covered by insurance for the subjects of this study, and are treatments that can be performed as part of routine insurance care. However, there is no evidence that the drugs used in this study will improve or prolong the prognosis of patients after preoperative chemotherapy. Since all medical expenses, including drug costs, during the study period for patients participating in the clinical study will be paid by the patient's insurance and patient co-payment, there is no special financial benefit to the patient for participating in this clinical study as compared to routine medical care.

**Anticipated risks and disadvantages**

All subjects will receive adjuvant therapy with nivolumab. This may increase the frequency of hematologic toxicities and infections, which can be considered an increased risk/disadvantage of participating in a clinical study.

In order to minimize the risk and disadvantage of these adverse events, "4.1 Selection Criteria" and "5.7 Criteria for Discontinuation of Protocol Therapy" are carefully reviewed. In the event of a serious or unexpected adverse event, a system is in place to ensure that any serious or unexpected adverse event is carefully reviewed and reviewed in accordance with the "JCTN - Adverse Event Reporting Guidelines" and related regulations, and that necessary measures are taken.

## 3.2 Anticipated illnesses, etc.

**Adverse Reactions Expected with Postoperative Nivolumab Therapy**

For expected adverse reactions, refer to the most recent version of the drug's package insert.

Drug inserts can be obtained from the search page of the Pharmaceuticals and Medical Devices Agency (PMDA).

# 4．Person who is being studied

Patients who meet all of the following selection criteria and none of the following exclusion criteria will be considered research patients for this clinical study.

## 4.1 Inclusion criteria：

Patients who meet all of the following criteria will be considered.

1. The primary lesion is located in the thoracic esophagus and is histologically diagnosed as squamous cell carcinoma by endoscopic biopsy.

2. Complete resection was performed after preoperative DCF (docetaxel, cisplatin, 5-FU) therapy, but no pathologic complete response was achieved.

3. The applicant is at least 18 years of age on the date of registration.

4. Gender is not required.

5. Performance status is 0 or 1 by ECOG criteria.

6. Patients can be enrolled between 4-16 weeks (28-112 days postoperatively) after complete resection.

7. Patients are confirmed disease-free by thoracoabdominal CT within 4 weeks prior to enrollment.

8. The most recent laboratory values within 14 days prior to registration (the same day of the week two weeks prior to the registration date is acceptable) meet all of the following

・ Absolute neutrophil count ≧ 1,500/mm^3^

・ Platelets ≧ 10.0×10^4^ /mm^3^

・ Hemoglobin ≧ 9.0 g/dL

・ Creatinine：No higher than the upper limit of normal × 1.5, or creatinine clearance ＞ 50mL/min (Cockcroft-Gault equation)

・ AST/ALT：No higher than the upper limit of normal × 3.0

・ Total bilirubin：No higher than the upper limit of normal × 1.5

9. The patient's free and voluntary written consent to participate in this clinical research has been obtained.

Reason for setting

1) It was set to ensure homology with the historical control JCOG1109 study.

2, 4, 6, 7) This was established to ensure homology with the inclusion criteria for the Checkmate-577 study.

3) The age at which an individual's consent is legally established was taken into consideration.

5) Set due to safety concerns at PS2 and above.

8) It was set to ensure safety.

9) Set for ethical considerations.

## 4.2 Exclusion criteria：

Patients with any one of the following will be excluded from the study

1. Infectious disease requiring systemic treatment.

2. Suffering from an active autoimmune disease.

3. Requirement for systemic steroid medication（≧ 10 mg/day of prednisolone）or immunosuppressants.

4. Currently or previously receiving treatment with immune checkpoint inhibitors.

5. Severe interstitial lung pneumonia or lung fibrosis.

6. The patient is deemed inappropriate by the person in charge of this clinical research.

7. Unwilling to use contraception and expecting and breastfeeding mothers.

Basis for setting

1-3, 5), 7) Set due to safety concerns.

　4) Set due to lack of expected effectiveness.

6) Established to leave room for the physician in charge to exclude ineligible patients in ensuring the safety of research subjects and in conducting clinical research properly.

# 5．Clinical Research Methods

## 5.1 Type and design

This clinical study is a single-arm, open-label, multicenter, phase II trial to evaluate the efficacy of nivolumab as adjuvant therapy in patients with thoracic esophageal squamous cell carcinoma who have failed to achieve a pathologic complete response to platinum-containing preoperative chemotherapy and who have had a complete resection at surgery following preoperative chemotherapy.

## 5.2 Outline

The duration of the clinical study consists of a 4-week pre-observation period, a maximum of 12 months during the study drug administration period, and a 4-year follow-up period. Study subjects will visit the hospital every 4 weeks during the study drug administration period, up to 12 months after the study drug administration (year 1). Study subjects will visit the clinic every 3 months from the end of study drug administration to 2 years after the start of study drug administration, and every 6 months in years 3-5. The study drug phase will consist of 480 mg of study drug (nivolumab) every 4 weeks for up to 12 months, administered on an outpatient basis.

## 5.3 How to register a case

### 5.3.1 Registration Procedure

The principal investigator or research associate physician will follow the procedure below from the time consent is obtained from the research subject through the initiation of protocol treatment.

1. After obtaining consent from the research subjects, the principal investigator or a research physician will conduct a screening test to determine the eligibility of the research subjects. If the consent of the research subject is obtained, the screening test may be used as a result of the tests required for enrollment, even if the tests were performed before consent was obtained.

If the consent of the research subject is obtained, tests performed prior to obtaining consent

may be used as necessary test results at the time of enrollment.

(2) After confirming that the research subject meets all of the selection criteria and none of the exclusion criteria, the principal investigator or research assistant investigator will access the EDC using the pre-issued user ID and password and enter the information necessary for case registration.

(3) The research office personnel will verify eligibility on the EDC, issue a case enrollment confirmation, and enroll the research subject. The principal investigator or research associate will confirm that the subject has been enrolled on the EDC and initiate protocol treatment.

(4) The principal investigator or subinvestigator shall print the "Case Registration Form" and "Case Registration Confirmation Form" from the EDC and store them appropriately as source documents.

### 5.3.2 Randomization Methods

This clinical study will not be randomized.

## 5.4 Protocol treatment

**Test Drug Administration Methods**

Since this product is an over-the-counter drug, it should be administered according to the

hospital's administrative procedures.

**Postoperative nivolumab therapy**

After enrollment, protocol treatment will be initiated within 14 days (same day of the week is allowed) and 480 mg of nivolumab will be administered as a 4-week course for up to 12 months from the start of protocol treatment until protocol discontinuation criteria are met. If it is determined that the first course cannot be started after 15 days, the details should be noted on the "End of Treatment Report Form" as "Protocol Treatment Discontinued".

**【Up to 12 months】**

| **Drug** | **Dose** | **Dosing time, method** | **Date of administration** |
| --- | --- | --- | --- |
| Nivolumab | 480 mg/day | IV infusion over 30 minutes | Every 4 weeks |

・The starting dose will be 480 mg/day for all patients, but drug withdrawal will be allowed in accordance with the criteria.

・In case of drug withdrawal, it should be noted in detail in the case report form

・If toxicity improves with adequate drug withdrawal, re-administration may be considered.

**・Protocol Treatment Initiation Criteria**

・Begin treatment after confirming that all of the following conditions are met on the day before or on the day of commencement.

・If the criteria are not met, do not administer the drug, delay administration until the criteria are met, and start administration as soon as the patient recovers. Once recovery is achieved, administration should be initiated.

**■Course Start Criteria**

① Absolute neutrophil count ≧ 1,500/mm^3^

② Platelets ≧ 10.0×10^4^ /mm^3^

③ Hemoglobin ≧ 9.0 g/dL

④ Creatinine：No higher than the upper limit of normal × 1.5, or creatinine clearance ＞ 50mL/min (Cockcroft-Gault equation)

⑤ AST/ALT：No higher than the upper limit of normal × 3.0

⑥ Total bilirubin：No higher than the upper limit of normal × 1.5

## 5.5 Regulations regarding concomitant medications

**Concomitant therapy**

The use of the following drugs is prohibited during the study administration period.

・ Immunosuppressive agents (excluding treatment for adverse events for which a causal relationship to the study drug cannot be ruled out)

・ Systemic corticosteroids with prednisone equivalents greater than 10 mg/day

・ Any combination of anti-tumor therapies (chemotherapy, hormonal therapy, immunotherapy, radiation therapy, or combination of any anti-tumor therapy (chemotherapy, hormonal therapy, immunotherapy, radiation therapy, or standard or investigational cancer therapy).

If the patient is on concomitant prohibited therapy, it must have been completed by the date of registration.

## 5.6 Drug Withdrawal Methods

**・Criteria for drug withdrawal and discontinuation**

If an adverse event is observed that meets the following criteria, the drug should be withdrawn or discontinued as follows.

If treatment cannot be resumed within 42 days of the last dose, protocol treatment is discontinued.

Contact the study office if you believe that drug withdrawal or discontinuation is necessary for an event not listed below.

**表5.6：Criteria for drug withdrawal and discontinuation**

|  | | **Interruption** | **Resuming administration** | **Discontinuation** |
| --- | --- | --- | --- | --- |
| **Interstitial pneumonia** | | Gr1 | Recovery to grade 0 | Grade2 or higher |
| **Colitis / Diarrhea** | | Gr2 | Recovery to grade1 or below | Grade3 or higher |
| **Liver dysfunction** | **AST/ALT** | Gr2 | Recovery to grade1 or below | Grade3 or higher |
|  | **T-Bil** | Gr2 | Recovery to grade1 or below | Grade3 or higher |
| **Thyroid dysfunction** | **Asymptomatic** | Continue to administration.  Consider starting hormone replacement therapy. | | |
|  | **Symptomatic** | Discontinue administration and start hormone replacement therapy. If the patients have recovered to grade 1 or below, consider resuming administration. | | |
| **Pituitary/adrenal dysfunction** | **Asymptomatic** | Continue to administration.  Consider starting hormone replacement therapy. | | |
|  | **Symptomatic** | Discontinue administration and start hormone replacement therapy. If the patients have recovered to grade 1 or below, consider resuming administration. | | |
| **Type 1 diabetes mellitus** | | Discontinue administration and start insulin replacement therapy. If the blood glucose levels has recovered to baseline, consider resuming administration. | | |
| **Kidney dysfunction：Creatinine** | | Gr2, Gr3 | Recovery to grade1 or below | Gr4 |
| **Neuropathy** | | Gr2 | Recovery to below baseline grade | Grade3 or higher |
| **Dermopathy** | | Gr3 | Recovery to grade1 or below |  |
| **Myocarditis / Myositis** | | Gr2 | Gr0 | Grade3 or higher |
| **Cerebritis** | |  |  | Grade3 or higher |

## 5.7 Criteria for discontinuation of protocol treatment

Protocol treatment will be discontinued if any of the following criteria are met. Even after discontinuation of protocol treatment, unless participation in the study is discontinued, investigation and observation as stipulated in the research plan will be conducted until the end of the follow-up period.

・ When the discontinuation criteria in Table 5.6 are met.

・ In case of recurrence.

・ When a research subject requests discontinuation of treatment due to illness or for other reasons.

・ Determination by the principal investigator or subinvestigator.

・ Death during protocol treatment.

・ Withdrawal of consent by research subject.

## 5.8 Medication Guidance Information

It is not an oral medication.

## 5.9 Deferred treatment

None/

## 5.10 After completion of the clinical research

This study concerns postoperative adjuvant therapy and the study subjects will not be affected by the results obtained after the study is completed.

# 6．Evaluation item

## 6.1 Primary endpoint

・Disease-free-survival（DFS）

## 6.2 Secondary endpoint

・Overall survival（OS）

・Distant metastasis-free survival（DMFS）

・Incidence of adverse events

### 6.2.1 Efficacy endpoints

**Disease-free-survival（DFS）**

Disease-free survival is defined as the period beginning on the date of registration and ending on the earlier of the date the cancer is determined to have recurred, the date the second cancer occurs, or the date of death from any cause.

- The program shall be terminated on the last day that the patient is confirmed to be disease-free (the final disease-free confirmation date).

- If information is obtained from a transfer or referring medical institution, receive and retain a medical information form with the basis for the diagnosis. Only telephone contact is not acceptable.

**Overall survival（OS）**

The period shall begin on the date of registration and end on the date of death from any cause.

- In survival cases, the last date of confirmation of survival shall be the date of termination.

- Untraceable cases are terminated at the last date of confirmed survival prior to the loss of follow-up.

**Distant metastasis-survival（DMFS）**

The period shall begin on the date of registration and end on the date of occurrence of the distant metastasis.

- The study shall be terminated on the last date when the patient is confirmed to be free of distant metastasis (the last date of confirmation of the absence of distant metastas

- Untraceable cases and deaths without a diagnosis of distant metastasis will be terminated as of the last date of confirmed distant metastasis prior to the loss of follow-up.

The last day of the follow-up period when the patient is confirmed to be free of distant metastasis before the loss of follow-up shall be the date of termination.

### 6.2.2 Safety endpoints

**Percentage of adverse even**

The frequency of the worst grade during the entire course will be determined using CTCAE v 5.0.

# 7．Observation and examination items

## 7.1 Implementation Schedule and Procedures

### 7.1.1 Schedule

| **Day**  **Item** | **Screening examination** | **Dosing period** | | **Follow-up period** | |
| --- | --- | --- | --- | --- | --- |
|  |  | **1 course** | **After 2 courses** | **Discontinuance** | **Follow-up survey** |
|  | **Before registration Within 14 days** | **Day 1** | **Day 1** |  |  |
| Treatment tolerance |  |  | ± 2 days | +14 days |  |
|  | **Examination finding** | | | | |
| Agreement | ○ |  |  |  |  |
| Patient background | ○ |  |  |  |  |
| Vital signs | ○ | ○ | ○ | ○ |  |
| Performance status（ECOG） | ○ | ○ | ○ | ○ |  |
| Symptoms | ○ | ○ | ○ | ○ |  |
| Adverse events |  | ○ | ○ | ○ | 〇 |
| Survival survey |  |  |  | 〇 | 〇 |
|  | **Clinical examination** | | | | |
| Hematological examination | ○ | ○ | ○ | ○ | ○ |
| Biochemical examination | ○ | ○ | ○ | ○ | ○ |
|  | **Oncological survey** | | | | |
| Computed tomography | ○ | Conducted once every 12 weeks. | | | |

### 7.1.2 Observation, examination and evaluation items

● **Screening**

After obtaining consent, the screening test is initiated. The principal investigator or research associate will perform the following screening tests. The tests will be as described below. In screening, if data from routine medical examinations or other data are available prior to obtaining consent, they may be used as research data.

1. Obtaining Consent：Date of consent, Subject Identification code
2. Background：Age, Date of birt, Sex, Medical history, Co-morbid disease, Date of surgery, Tumor location, Resection and reconstruction, Chest operation method, Abdominal operation method, Reconstructed organ, Route of reconstruction, Anastomotic method, Range of lymphnode dissection, Operation time, Chest operation time, Blood loss, Combined excision, Perioperative transfusion, Postoperative complications, TNM classification, Number of cervical lymph node metastases, Number of mediastinal lymph node metastases, Number of abdominal lymph node metastases, Metastatic lymph node site, Tissue differentiation degree, Vein invasion, Lymphatic invasion, Number of preoperative chemotherapy, Efficacy rate of pathology, Multiple cancers
3. Vital signs (pulse, blood pressure, temperature, SpO2)
4. Performance status（ECOG）
5. Symptoms
6. Hematological examination
7. Biochemical examination（AST, ALT, ALP, LDH, T-BIL, BUN, Cr, Na, K, CI, Ca, Glu, Alb,

Amy, LIPA, HbA1c, TSH, FT4, ACTH, Cortisol)

1. Thorax and abdomen CT: Confirmation of disease-free

**● During treatment, up to 12 months**

**Day 1 of each course**

1. Vital signs (pulse, blood pressure, temperature, SpO2)
2. Performance status（ECOG）
3. Symptoms
4. Adverse Event Assessment
5. Hematological examination
6. Biochemical examination（AST, ALT, ALP, LDH, T-BIL, BUN, Cr, Na, K, CI, Ca, Glu, Alb,

Amy, TSH, FT4)

1. Thorax and abdomen CT:：Once every 12 weeks.

**● During the follow-up period**

If protocol treatment is discontinued due to recurrence, only tumor evaluation and a survival study at 5 years will be performed.

1. Adverse Event Assessment
2. Hematological examination
3. Biochemical examination（LDH, AST, ALT, ALP, T-BIL, BUN, Cr, Na, Ca, K, CI, Glu, Alb,

Amy)

④ Thorax and abdomen CT:：Once every 12 weeks.

1. Survival survey

**● Discontinuance**

1. Vital signs (pulse, blood pressure, temperature, SpO2)
2. Performance status（ECOG）
3. Symptoms
4. Adverse Event Assessment
5. Hematological examination
6. Biochemical examination（AST, ALT, ALP, LDH, T-BIL, BUN, Cr, Na, K, CI, Ca, Glu, Alb,

Amy, TSH, FT4)

1. Thorax and abdomen CT:：Once every 12 weeks

## 7.2 Inspection contractor

In this clinical study, measurement items will be measured at the collaborating institutions listed below.

**（Measurement items and measurement institutions）**

| **Item** | **Measuring agency or method** |
| --- | --- |
| TPS | Measurement company：LSI Medience Inc. |
| CD103^+^CD8^+^Tcell, Foxp3^+^CD4^+^Tcell, Bcl6^-^CD4^+^Tcell | Measurement company：Kobe University Hospital  Method：AI Curation Association.  AI pathology system CuCyte used |

## 7.3 Compliance with research protocols and procedures

### 7.3.1 Compliance with research protocols and procedures

The Principal Investigator or the Research Assigning Physician shall conduct the research in compliance with the research protocol, each procedure, the Clinical Research Act, and the implementing regulations, except for unavoidable reasons such as emergency avoidance.

Research subjects will comply with this research plan as long as their safety and human rights are not compromised. Personnel engaged in this research will properly disclose the results of the research in accordance with the "Guidelines on Responses to Misconduct in Research Activities" and the regulations of each medical institution.

### 7.3.2 Critical Nonconformity

A serious noncompliance is defined as a condition in which the clinical research does not conform to the research protocol, etc., which affects the human rights and safety of the subjects of the clinical research as well as the progress of the research and the reliability of the results (such as non-compliance with selection/exclusion criteria, discontinuation criteria, concomitant use of prohibited therapies, etc.).

However, noncompliance with the research protocol, etc., in order to avoid immediate danger to clinical research subjects or for other unavoidable medical reasons will not be considered a major noncompliance.

### 7.3.3 Procedures in case of non-compliance with the research protocol, etc.

When the research associate physician learns that the clinical research is noncompliant, he/she shall promptly report it to the principal investigator.

When the principal investigator learns that the clinical research is noncompliant, he/she shall promptly report this to the administrator of the implementing medical institution and notify the principal investigator of the noncompliance. However, if there is concern that reporting to the principal investigator will result in a failure to report to the administrator of the implementing medical institution, the research physician should report directly to the administrator of the implementing medical institution. When the notified principal investigator learns that the clinical research is noncompliant, he/she will promptly inform the other principal investigators of the noncompliance. If a serious noncompliance is identified, the Principal Investigator will ask the Accredited Clinical Research Review Committee for its opinion.

# 8．Criteria for discontinuance

If the principal investigator or subinvestigator determines that the clinical research cannot be continued for any of the reasons listed below, the investigator or subinvestigator will discontinue the subject's participation in the research, and the date and time of discontinuation or dropout, the reason for discontinuation or dropout, and the progress will be noted in the medical record and CRF, and necessary tests will be performed at the time of discontinuation or dropout to evaluate efficacy and safety.

**〈Criteria for discontinuance〉**

- - 1. When a research subject declines to participate in clinical research or withdraws consent.
    2. Eiigibility is not satisfied after registration.
    3. When side effects make it difficult to continue.
    4. When pregnancy is detected.
    5. Significant non-compliance.
    6. When the entire clinical study is terminated.
    7. When the principal investigator or a subinvestigator determines that it is appropriate to discontinue the clinical research for other reasons.

# 9．Handling of Adverse Events

## 9.1 Definition.

## 9.1.1 Adverse events

Adverse events are defined as "illness, disability, death, infection, abnormal laboratory values or symptoms" that occur during participation in this clinical research. No causal relationship to this clinical research is involved.

## 9.1.2 Diseases, etc.

Disease" means an adverse event that is suspected to be attributable to the conduct of this clinical research participation.

## 9.1.3 Serious adverse events, illnesses, etc.

Serious adverse events or illnesses are those falling under any of the following (1) to (3).

① Death

② Diseases that could lead to death

③ Illness that requires hospitalization or extended stay in a medical institution for treatment

④ Disability

⑤ Diseases that may lead to disability

⑥ Diseases that are serious according to (1) to (5)

⑦ Congenital diseases or anomalies in later generations

## 9.1.4 Unanticipated adverse events and illnesses

Unanticipated adverse events or illnesses, etc., are those whose occurrence is not described in the research protocol, the drug's abstract, or the patient information document.

1. Items that cannot be predicted from the directions for use on the package insert or on the container or packaging of the drug or other products used in clinical research.
2. Items for which the trend of occurrence cannot be predicted based on the precautions for use of the drug, etc. in question.
3. The one that can be predicted from the precautions for use of the drug concerned, etc., and the change in the trend of occurrence indicates the occurrence or threat of expansion of a health hazard.

## 9.2 Adverse events to be collected

The principal investigator or research associate will report in the case report all adverse events that occur from the time the study drug is administered until 30 days after the end of study drug administration, and will observe until 8 weeks after the end of participation in the clinical study to see if the adverse events have resolved. All illnesses, etc. suspected to be caused by the study drug or this clinical research will be reported until the end of the clinical research period.

## 9.3 Description required for evaluation of adverse events

When an adverse event occurs, the following items should be noted in the case report form.

1)　Name of adverse event

2)　Expression date

3)　Day of reflections

4)　Outcome：Recovered, mildly recovered, with sequelae, unrecovered, dead, unknown

5)　Administration of study drug：No change, discontinuation, withdrawal, dose reduction, dose increase, not applicable

6)　Other treatment：None, drug treatment, other

7) Severity：Non-serious, serious

8)　Severity Level：Mild, moderate, severe

- 1. Mild：Condition that allows administration to continue without treatment.
  2. Moderate：Condition that allows continuation of administration by some measures.

③　Severe：Conditions that should result in discontinuation of dosing or discontinuation of the clinical study.

9) Causal relationship with clinical research (applicability to diseases, etc.): Related, not related

10) Causal relationship with study drug: related, not related

### 9.3.1 Causal relationship between disease recovery and clinical research

Recovery of disease is defined as the absence of disease or improvement to the state before administration. In determining whether a disease is suspected to be caused by the clinical research, the investigator will take into consideration the investigator's general condition, complications, concomitant medications/adjunctive therapies, and temporal relationship.

### 9.4　Provision of medical care

If an adverse event occurs during the conduct of clinical research, the principal investigator or research assistant who has knowledge of the adverse event will provide appropriate medical care to the subject of the clinical research regarding the adverse even. The principal investigator or research physician will provide appropriate medical care to the subject of the clinical research until the subject recovers to the condition before the adverse event or stabilizes symptoms, and will follow the subject until it is determined that follow-up is no longer necessary.

## 9.5 Procedures in case of disease outbreak

Procedures in the event of disease outbreaks shall follow the "Procedures for Handling Disease Outbreaks.

# 10．Initiation, discontinuation, suspension and termination of clinical research

## 10.1　Initiation of clinical research

At the start of this clinical research, the implementation plan will be registered with the Japan Registry of Clinical Trials and made publicly available after obtaining the opinion of the Accredited Clinical Research Review Committee. The date of registration and publication of the study information in jRCT shall be the study start date. The principal investigator will provide information to the accredited clinical research review committee that heard the opinion and to the other principal investigators when the information is released to the jRCT. Upon receiving the information, the principal investigator reports to the administrator of each institution. No study subjects will be enrolled prior to publication in jRCT.

## 10.2 Criteria for suspending or terminating an entire clinical study

The principal investigator will consult with the other principal investigators and decide on the suspension or discontinuation of the entire study when the following information is obtained and it is considered difficult to continue the entire study.

i) When the following information is obtained during this clinical research.

- 1. Unexpected occurrence of serious adverse reactions
  2. Information indicating that trends in the occurrence of predictable serious adverse reactions, including the number of occurrences, frequency of occurrence, and conditions of occurrence, cannot be predicted from the interview form and accompanying medical information.
  3. Information on serious diseases for which no causal relationship was determined to exist, but for which a causal relationship is subsequently determined to be undeniable based on trends in the number of occurrences, frequency of occurrences, and occurrence of conditions.

ii) When any of the following information regarding the study drug in this clinical research is made public.

1. Research reports indicating a significant change in the number of adverse reactions, frequency of occurrence, conditions of occurrence, or other trends in the occurrence of adverse reactions.
2. Research reports indicating a risk of cancer or other serious disease, disability, or death
3. Information indicating that the product is not indicated or effective for the disease that is the subject of this clinical research
4. Information on the implementation of measures to prevent the occurrence or spread of health hazards, such as discontinuation of manufacture, import, or sale, recall, or disposal of over-the-counter drugs containing the same ingredients as the study drug.

## 10.3　Procedures for suspending an entire clinical study

### 10.3.1 Procedures for suspending an entire clinical study

If the principal investigator discontinues the clinical research after consultation with other principal investigators, the principal investigator shall submit changes to the implementation plan to the Accredited Clinical Research Review Committee without delay.

The research subjects participating in the study shall be promptly informed and appropriate action taken to change to the appropriate treatment.

### 10.3.2 Procedures for Resumption of Clinical Research

Before resuming the interrupted clinical research, the Principal Investigator shall review the need to revise the research protocol and each procedure, and if changes are necessary, they shall be made in accordance with "Changes to Research Protocol, Procedures, or Statistical Analysis Protocol".

The Principal Investigator submits a change in the research plan for resumption of the research to the Accredited Clinical Research Review Committee.

## 10.4　Procedures for discontinuation of clinical research

### 10.4.1 Procedures for discontinuation of the entire clinical research

If the principal investigator, after consultation with the other principal investigators, discontinues the entire clinical research, the principal investigator will notify the Accredited Clinical Research Review Committee within 10 days of the discontinuation.

Promptly inform the research subjects participating in the clinical research of the discontinuation of the clinical research and take appropriate measures such as changing to appropriate treatment.

The principal investigator shall submit notifications such as changes in the implementation plan, disease reports, and periodic reports in the same manner as during the conduct of the research until the clinical research is terminated after all subjects have been observed from the time of discontinuation of the clinical research.

The Principal Investigator shall terminate the clinical research in accordance with "Termination of Clinical Research" within one year from the date of discontinuation of the clinical research or completion of all subject measures, whichever is later.

### 10.4.2 Procedures for discontinuation of clinical research at individual institutions

When a principal investigator discontinues clinical research at his/her institution, he/she shall promptly notify the principal investigator in writing to that effect and report to the administrator of the institution in accordance with the regulations of the institution.

When the Principal Investigator receives a report of discontinuation of clinical research at an individual institution, he/she will submit to the Accredited Clinical Research Review Committee a revised implementation plan involving the institution after completing observation and other measures for the subject at the institution.

The principal investigator will inform the other investigators of the change in the site.

## 10.5 Completion of clinical research

The principal investigator shall prepare a summary report and a summary of the summary report, in principle, within one year from the date of completion of the period for collecting data on all endpoints. After preparing the summary report, submit it without delay together with the notice of termination and submit it to the Accredited Clinical Research Review Committee for its opinion without delay.

The principal investigator shall publish a summary of the summary report in jRCT within one month of the date on which the accredited clinical research review committee gives its opinion. The clinical research shall be terminated on the date of publication of the summary of the overall report.

The principal investigator will report the termination of the clinical research to the administrator of the implementing medical institution and provide information to other principal investigators. The principal investigator reports the details of the information provided to the administrator of the implementing medical institution.

# 11．Clinical Research Period

Date of publication of implementation plan to July 31, 2031

Registration Period: Date of publication of implementation plan to July 31, 2031

Follow-up period: 5 years from the date of registration

# 12．Analysis object, and statistical analysis method

The following is a summary of the statistical analysis plan for this clinical study.

If the analysis plan has been modified from the original plan, this should be explained in the summary report.

## 12.1 Population to be analyzed

### 12.1.1 Largest analyzed population (full analysis set：FAS)

Consists of study subjects who have received at least one dose of study drug and whose efficacy has been evaluated at least once among all enrolled subjects.

### 12.1.2 Analysis population conforming to the research protocol (per protocol set：PPS)

FAS, excluding cases of the following serious violations of the provisions of the research protocol in the clinical research methods or concomitant therapies. The purpose of this study is to confirm the stability of the primary analysis for the efficacy endpoints.

・ Selection criteria violation

・ Exclusion Criteria Violations

・ Significant noncompliance with the study protocol that could affect the assessment of efficacy

### 12.1.3 Safety analysis population (Safety Analysis Set: SAS)

Patients enrolled in this clinical study and who have received the study drug at least once will be included in the analysis.

## 12.2 Number of cases and rationale for setting

Number of cases：130 cases

| **Name** | **Planned number of cases** |
| --- | --- |
| Kobe University Hospital | 40 cases |
| Hyogo Cancer Center | 30 cases |
| Kakogawa Central City Hospital | 20 cases |
| Saiseikai Nakatsu Hospital | 5 cases |
| Kitaharima Medical Center | 20 cases |
| Awaji Medical Center | 5 cases |
| Harima-Himeji General Medical Center | 5 cases |
| Meiwa Hospital | 3 cases |
| Konan Medical Center | 2 cases |

The number of expected cases at each facility is only a guideline, and the number of expected cases may be increased or decreased depending on the status of case enrollment. The number of cases for each facility will be specified in the summary report. The primary analysis objective of this clinical study is to determine whether the protocol treatment arm (postoperative nivolumab treatment) outperforms the historical control (DCF treatment arm of JCOG 1109) in the primary endpoint, 3-year DFS.

If the protocol treatment group statistically significantly outperforms the historical control, we conclude that the protocol treatment, preoperative adjuvant therapy with DCF plus postoperative adjuvant therapy with nivolumab, is the more useful treatment. If not significantly superior, conclude that preoperative adjuvant therapy with DCF, the standard of care, remains a useful treatment.

In this clinical study, if the protocol treatment group is inferior to the historical control group, the test is a one-tailed test because it is not of interest whether it is statistically significant or not, and the significance level for the entire study is 5% one-sided. The 3-year recurrence-free survival rate for DCF preoperative adjuvant therapy plus nivolumab postoperative adjuvant therapy is expected to be 74%, 10% higher than the 64% 3-year recurrence-free survival rate for historical control. Assuming a 3-year enrollment period and 5 years of medication and follow-up, at least 123 patients would be needed for a survival time analysis with 80% power. Based on the above, the target number of patients for this clinical study was set at 130, assuming a dropout rate of 10% and a small number of ineligible cases.

## 12.3 Case Handling

In principle, the principal investigator and the statistical analyst will decide on the handling of the registered cases after consultation with the principal investigator and the statistical analyst. The principal investigator and the statistical analyst will also decide, after consultation, how to handle cases in case new problems arise.

## 12.4 Data Handling

### 12.4.1 Handling of Data

If any questions arise regarding the handling of data during data aggregation and analysis, the Principal Investigator and the Chief Statistical Analyst will discuss and make a decision. Procedures for handling missing, rejected, and abnormal data will be decided upon consultation between the Principal Statistical Analyst and the Principal Investigator.

### 12.4.2 Data collection after discontinuation of the study

For research subjects who are discontinued during the course of the study, data collected up to the point of discontinuation will be used as research data. However, this does not apply when there is a withdrawal of consent for the use of data from the research subject.

## 12.5 Statistical analysis items and analysis plan

Analysis will be performed after study drug administration has been completed in all cases and data have been fixed. For all efficacy evaluations, the analysis in the FAS will be the primary analysis and the analysis in the PPS will be performed as a reference. The safety analysis will be performed in SAS.

### 12.5.1 Aggregation of Study Subject Backgrounds

・Calculate the distribution and summary statistics of the study subject background data for each analysis population.

・For nominal variables, the frequency and percentage of categories are shown.

・For continuous variables, summary statistics (number of cases, mean, standard deviation, minimum, median, maximum) are calculated.

### 12.5.2 Efficacy Analysis

**12.5.2.1 Indicators for validity assessment**

**3-year disease-free survival rate**

Definition：The disease-free survival rate at 3 years (365.25 x 3 days) estimated using the Kaplan-Meier method is the 3-year disease-free survival rate.

**12.5.2.2 Principal Analysis**

The primary objective of this clinical study is to evaluate the efficacy of adjuvant nivolumab in patients with squamous cell carcinoma of the esophagus who underwent esophagectomy after preoperative chemotherapy with DCF at the 3-year disease-free survival rate, the primary endpoint of this clinical study. No simultaneous contrast is set.

The 3-year disease-free survival rate is calculated using the Kaplan-Meier method for all eligible cases, and the 90% and 95% confidence intervals of the point estimates are calculated using the Greenwood formula. Survival time analysis with the null hypothesis being "the survival rate at 3 years is less than 64%" with a one-sided significance level of 5% for the test.

**12.5.2.3 Subanalysis**

Analysis of secondary efficacy endpoints for the purpose of providing additional discussion to supplement the results of the primary analysis. The 5-year overall survival and 3-year metastasis-free survival rates are calculated using the Kaplan-Meier method for all eligible cases, and the 95 % confidence interval of the point estimate is calculated using the Greenwood formula.

### 12.5.3 Safety Analysis

**12.5.3.1 Safety Assessment Indicators**

　Incidence of Disease

**12.5.3.2 Safety Analysis**

The safety endpoint is the frequency of occurrence of disease, etc. Tables are prepared for the endpoints, and exact two-sided 95% confidence intervals of binomial distribution are calculated for percentage estimation.

### 12.5.4 Interim Analysis

No interim analysis will be performed in this clinical study.

**12.5.5 Supplementary analysis**

　　　　Analysis by subpopulation by expression of CD103+CD8+Tcell, Foxp3+CD4+Tcell, and Bcl6-CD4+Tcell was performed for the primary endpoints and secondary endpoints. Survival time analysis using the COX proportional hazards model was performed for each T-cell expression status. The significance level of the test is 5% two-sided.

## 12.6 Independent Data Monitoring Committee

This study will not establish an independent data monitoring committee.

**12.7 Final Analysis**

After the follow-up period, analysis will be performed after data are obtained and cases are fixed. The person responsible for statistical analysis will compile the "Statistical Analysis Report" and submit it to the principal investigator.

# 13．Changes in research protocols, procedures, or statistical analysis plans

Follow this procedure when making changes or additions to the following documents in this clinical research.

- Implementation Plan（jRCT）
- Research plan
- Description
- Consent document
- List of Research Physicians
- Procedures for responding to outbreaks of disease
- Monitoring Procedures
- Contrary interest management basis
- Contrary interest management plan
- Documents describing the outline of the pharmaceutical product
- Key Evaluative Items Report (when newly created or modified)
- Other

If the Principal Investigator deems it necessary to revise or create a new document, he/she will provide the Principal Investigator with a revised draft of the research protocol and the most recent attached documents.

The Principal Investigator will give the investigator sufficient time to review the materials and information provided by the Principal Investigator in accordance with the preceding paragraph, including the proposed revisions to the research protocol, and to discuss the proposed revisions with the Principal Investigator before finalizing the revisions.

The Principal Investigator submits the document to be revised or newly created and the application for change to the Authorized Clinical Research Review Committee, obtains the opinion and approval of the Authorized Clinical Research Review Committee, and then reports to the Administrator of the Operating Medical Institution.

The Principal Investigator will make changes to the jRCT if they involve changes to the implementation plan.

The principal investigator will inform the other principal investigators of any changes in the research protocol.

The principal investigator reports the content of the information provided to the administrator of the implementing medical institution.

The Principal Investigator shall notify the Accredited Clinical Research Review Committee of any minor changes in the implementation plan within 10 days of the date of the change

Minor changes

・A change in the name or contact information of a person engaged in the specified clinical research or the name of the organization to which such person belongs, which does not involve a change in such person or the organization to which such person belongs.

・Change of contact point for receiving complaints and inquiries.

・Change of the name of the administrator of the institution to which the principal investigator or principal study authorization holder belongs.

・Changes associated with the administrator's approval or disapproval to conduct a specific clinical research study.

・Changes in matters relating to confirmation of the status of the conduct of the specified clinical research that do not involve changes in the results of the clinical research and the conduct of the audit.

・Change in the name or contact information of the Accredited Clinical Research Review Committee that performs review and opinion services that does not involve a change in the said Accredited Clinical Research Review Committee.

・Other

# 14．Data management

## 14.1 Creating a Case Report Form (CRF)

The principal investigator or research associate will enter case report data for each research subject into EDC (Electronic Data Capture).

When inputting data, access the EDC using the pre-issued user ID and password, and enter or modify the data while referring to the EDC's operation manual and input manual.

After confirming that there are no errors in the prepared CRF, the principal investigator electronically signs the CRF on the EDC. The signed CRF should be kept for archival purposes. Export for archival purposes and store on external media.

When an inquiry is received from the data center regarding the input contents of the CRF, check the EDC and respond promptly. If the inquiry form issued by the data center is in paper form, it should be stored appropriately with the medical record.

## 14.2 Identification of source materials

The following documents and other materials will be used as source documents in this clinical research.

1)　Records of consent of research subjects and provision of information to research subjects.

2)　The record on which the case report was based.

Data stored in electronic medical records are also considered source documents.

3)　Records of study drug administration.

4)　Documents or records required under the Clinical Research Act pertaining to this clinical research.

Of the data described in the case report form, the following items shall be considered as source documents when they are described in the case report form. However, if the data is recorded in the medical record, the medical record shall be considered as the source data.

1)　Disease severity, outcome, severity, determination of causal relationship to this clinical study and basis for determination.

2)　Reason for discontinuation of clinical research on research subjects.

3)　Comments by principal investigator or research associate.

# 15．Quality Control and Quality Assurance

## 15.1 Quality control

From the perspective of ensuring the reliability of this clinical research and protecting research subjects, monitoring will be conducted with the objective of periodically confirming that the research is being conducted in accordance with the relevant regulations such as the Clinical Research Act and the research protocol.

### 15.1.1 Designation of Monitoring Manager and Monitor

The Principal Investigator shall designate the person responsible for monitoring and the person in charge of monitoring for the study. The person in charge of monitoring and the monitor shall be appointed from among those who have educational backgrounds regarding the "Clinical Research Act" and other regulatory requirements, and who fully understand the contents of the research protocol of this clinical research, the research subject's explanatory consent document, and the monitoring procedure manual.

### 15.1.2 Monitoring

**15.1.2.1 Monitoring of cases**

Throughout the period in which the clinical research is being conducted, the monitors will analyze and evaluate the materials and research data submitted to the Research Office and others, and monitor based on this information. Items to be checked are specified in the "Procedures for Monitoring Implementation.

**15.1.2.2 Non-Case Monitoring**

Monitors will conduct monitoring before the start of the clinical study, during the study, and after the study is completed. Items to be checked in monitoring are specified in the "Procedures for Conducting Monitoring.

## 15.2 Quality assurance

This study will not be audited.

## 15.3 Direct acess to oiginal mterials

The Principal Investigator and the Performing Healthcare Organization will provide direct access to all original clinical research-related records during monitoring related to the clinical research and during investigations by the Accredited Clinical Research Review Board and regulatory authorities.

# 16．Compliance with the Clinical Research Act and the Declaration of Helsinki

This clinical research will be conducted in compliance with the declaration of Helsinki, the clinical research act, the conflict of interest management plan for this clinical research, and the personal information protection act.

# 17．Consideration for human rights, safety and disadvantage of research subjects

## 17.1 Consideration for Human Rights (Protection of Personal Information)

Personal information necessary for this clinical research will be obtained with the consent of the research subjects. Personal information obtained will not be used for any purpose other than to conduct this clinical research and any research that may occur later.

In order to handle personal information appropriately, the principal investigator will be responsible for managing personal information in this clinical research as the responsible party at the relevant medical institution. Principal investigators and subinvestigators will follow the rules for the handling of personal information at the relevant medical institution. If such regulations are not in place at the implementing medical institution, the "Internal Rules on Measures for the Appropriate Management of Personal Information Related to Medical Care Held by Kobe University Hospital" of Kobe University Hospital, which is the principal research facility, will be followed.

In the event of leakage, loss, or damage of personal information, the principal investigator shall promptly report the incident to the principal investigator and the personal information protection manager of the medical institution concerned, and take appropriate measures.

All parties involved in this clinical research will adhere to the principle of protecting the rights of research subjects against invasion of their privacy. The utmost efforts will be made to protect the personal information and privacy of research subjects, and personal information obtained in the course of conducting this clinical research must not be divulged without justifiable reason. The same shall apply after the person concerned has retired from position. In this clinical study, a list of subject identification codes will be used to associate the clinical research database and clinical research-related documents with the original data of the research subjects. The principal investigator will keep the list of subject identification codes in a lockable location and separate from other documents related to this clinical research.

Limited research subject information, such as gender, date of birth, etc., may be used to identify research subjects and to verify the accuracy of the research subject identification code list, within the limits of all applicable laws and regulations.

Personnel involved in this clinical research will use the research subject identification code when managing data. When providing samples and information to other principal investigators, some (name, personal identification code (hospital ID)) or all of the personal information will be deleted. The principal investigator will not provide the list of research subject identification codes outside the respective institution.

When the principal investigator releases information obtained through clinical research, sufficient care should be taken to ensure that the research subjects cannot be identified.

## 17.2 Provision of samples and information to other institutions

### 17.2.1 Provision of samples and information to other institutions

This clinical research is a multicenter collaborative study, and the samples and information collected in accordance with "7.1, Implementation Schedule and Procedures" will be used jointly within the scope specified in "25 Research Organization. Case reports from each practicing institution will be sent to the research office after removing some of the personal information. The list of research subject identification codes will be kept at each implementing medical institution and will not be provided to outside parties.

In this clinical study, TPS testing was contracted to LSI Medience Inc. The information related to the test will be sent to LSI Medience Inc. with all personal information deleted, and the information will be sent to LSI Medience Inc. in a form that retains a record of the sending of the information.

### 17.2.2 Provision of samples and information to persons in foreign countries

Not applicable.

## 17.3 Burden and foreseeable risks to research subjects

### 17.3.1 Projected Profits

The drugs used in this clinical study are approved and covered by insurance for the subjects of this clinical study, and are treatments that can be performed as routine insurance treatment. However, there is no evidence in postoperative patients after preoperative chemotherapy, and the administration of the drugs used in this clinical study is expected to benefit patients by improving and prolonging their prognosis. Since all medical expenses, including drug costs, during the study period for patients participating in the clinical study will be paid by the patient's insurance and patient co-payment, there is no special financial benefit to the patient for participating in this clinical study as compared to routine medical care.

### 17.3.2 Anticipated disadvantages

By participating in this clinical study, the occurrence of diseases as described in "3.2 Expected Diseases" is expected to be higher than in the usual medical treatment.

## 17.4 Safety and disadvantage considerations

In order to minimize the risk of disease and other disadvantages incurred in this clinical research, "4.1 Selection Criteria" and "5.7 Criteria for Discontinuation of Protocol Treatment" have been carefully considered. In addition to monitoring whether or not the disease outbreaks are within the expected range, any serious or unexpected disease outbreaks are carefully examined and reviewed, and necessary countermeasures are taken. As a rule, the cost of treatment provided for other health hazards is paid by health insurance and patient contributions.

## 17.5 Report to the Accredited Clinical Research Review Committee

### 17.5.1 Periodic report

The Principal Investigator shall report in writing to the Administrator of the site and to the Accredited Clinical Research Review Committee every year from the date the implementation plan is published in the jRCT, regarding the following.

The principal investigator will provide information on the content of the periodic report to the other principal investigators. Each principal investigator will report the content of the information provided to the administrator of the implementing medical institution.

- 1. Number of research subjects who participated in this clinical research and the number of reports of illnesses, etc. to the authorized clinical research review committee.
  2. Incidence and subsequent progress of diseases related to this clinical research
  3. Status of occurrence of serious noncompliance with respect to this clinical research and subsequent actions.
  4. Evaluation of the safety and scientific validity of this clinical research.
  5. Status of conflict of interest management.

### 17.5.2 On-the-spot report

The Principal Investigator will report to the Administrator of the site and the Accredited Clinical Research Review Committee whenever he becomes aware of a reportable disease, a serious noncompliance, or when the study is terminated. The principal investigator will provide information to the other principal investigators when he/she makes the aforementioned report. Upon receiving the information, the principal investigator will report to the administrator of the respective institution.

## 17.6 Approach to disclosure of information on genetic mutations and contingent findings

No human genome or genetic analysis will be performed in this clinical study.

## 17.7 About Genetic Counseling

Since this clinical study will not involve human genetic analysis, genetic counseling will not be provided.

## 17.8 Secondary use of data and samples

The data of the study subjects obtained in this clinical study will be compiled into a database, which may be used for other studies in the future. When conducting other research, a new research plan should be developed and approved by the subject's Ethics Review Committee. In such cases, we will not seek written consent from individual research subjects, but we will disclose information about the research in an appropriate manner.

# 18．How to explain to research subjects and obtain their consent

## 18.1 Subject of consent

Consent for this clinical research will be obtained from the research subjects themselves, and consent will not be obtained from a surrogate.

## 18.2 Preparation and revision of consent and other explanatory documents

The principal investigator will prepare the written consent and other explanatory documents used to obtain consent for participation in the research from the research subjects, using plain language to the greatest extent possible. When information is obtained that may influence the intention of the subject of the clinical research or the surrogate regarding continued participation in the research, the explanation and consent document and other explanatory documents will be revised promptly. The procedure for revision shall be in accordance with "Changes in Research Protocols, Procedures, or Statistical Analysis Plans".

## 18.3 Procedures for obtaining consent from research subjects

The Principal Investigator or Research Assigning Physician will provide explanations to the potential research subjects using the explanatory documents accompanying this research protocol. The principal investigator or research associate will give the potential research subject an opportunity to ask questions and sufficient time to decide whether or not to consent, and will obtain free and voluntary consent in writing after confirming that the potential research subject has a good understanding of the content of this clinical research.

The principal investigator or research associate will obtain re-consent from the research subjects when the informed consent document and other explanatory documents are revised in a manner that may affect the research subjects' willingness to participate.

## 18.4 Procedures for obtaining informed consent from a substitute or other party

Not applicable.

## 18.5 Procedures for receiving informed assent

Not applicable.

## 18.6 Explanatory notes for research subjects

The following items should be included in the letter of explanation prepared by the principal investigator. The details are described in the attached explanation and consent document.

- 1. Introduction.
  2. Implementation of clinical research.
  3. Participation in clinical research.
  4. The medical conditions and treatment of the patients in this clinical study.
  5. Significance and purpose of this clinical study.
  6. the methodology of this clinical study.
  7. Schedule and implementation matters.
  8. Medical institutions conducting this clinical research and the number of expected participants.
  9. After the completion of the clinical research.
  10. Expected benefits and expected disadvantages.
  11. When your health is harmed during this clinical research.
  12. When new information about this clinical research becomes available.
  13. Other treatment methods
  14. Discontinuation of clinical research.
  15. Fees and honoraria related to this clinical research
  16. Method of storage and disposal of samples
  17. Access to medical records and protection of personal information.
  18. Information disclosure regarding clinical research.
  19. Handling of research data.
  20. Ownership of intellectual property rights
  21. Sources of funding and conflicts of interest related to the clinical research in question.
  22. What we ask you to observe.
  23. Contact for clinical research inquiries.

# 19．Compensation for health hazards

This clinical study will use drugs already approved by the MHLW. Therefore, if any adverse health effects are caused by the treatment related to this clinical research, the principal investigator or subinvestigator of this clinical research will be responsible for treatment, and no financial compensation will be provided for such effects. In the event of any failure, the research subjects will be promptly treated with appropriate measures and therapy, but the cost of such therapy will be borne by the research subjects themselves, as it will be covered by their regular insurance.

## 19.1 Indemnification by Clinical Research Insurance

To ensure that appropriate action can be taken in the event of a claim from a research subject for health damage incurred in connection with the conduct of this clinical research, the company will purchase a clinical research insurance policy that provides compensation for death, serious disability, medical expenses and medical benefits.

## 19.2 Non-Clinical Research Insurance

Not applicable.

# 20．Cost sharing for research subjects

All drugs used in this clinical study will be covered by insurance. However, the TPS examination, which is an evaluation item, will be funded by the research fund of the department to which the principal investigator belongs. Since the number of hospital visits will not increase compared to regular medical care, there will be no additional cost burden as a result of participation in the clinical research.

# 21．Preservation of recorded documents

## 21.1 Preparation of records related to clinical research

The principal investigator of each medical institution shall, in accordance with the Clinical Research Act, prepare a record of the date, time, and place of use of pharmaceutical products, etc., for each subject, matters identifying the subject, matters concerning medical treatment and examination of the subject, matters concerning participation, and matters necessary for the implementation of the study.

## 21.2 Record keeping for clinical research

The principal investigator at each site will appropriately keep records related to this clinical research and the following documents in accordance with the arrangements at each site. The period of storage shall be the period specified by each institution or five years from the date of completion of this clinical research, whichever is longer. After that, the data will be disposed of in a state in which individuals cannot be identified.

1. Research plan and implementation plan
2. Documents related to the explanation to the research subjects and their consent
3. General report
4. Documents pertaining to review opinion services received from an accredited clinical research review committee
5. Documents related to monitoring
6. Original documents
7. Contracts related to this clinical research
8. Documents describing an outline of the drugs, etc. used in the clinical research
9. Other documents or copies thereof prepared by the principal investigator in accordance with the clinical research act and its implementing regulations
10. Samples, equipment, etc. related to this clinical research

## 21.3 Correction of records related to clinical research

If the principal investigator amends the records created, he/she shall record the name of the person who made the amendment and the date the amendment was made, and retain the amended records together with the amended records.

If records obtained from research subjects or case reports are changed or modified, the reason for the change or modification and the history of the change or modification should be recorded.

# 22．Register a research plan

This clinical study will be enrolled in jRCT based on the recommendations of the International Committee of Medical Journal Editors. The plan will be updated as necessary according to changes in the implementation plan and the progress of the study.

# 23．Disclosure of research funding sources and conflict of interest status

**23.1 Sources of funding for research**

This study used Opdivo, a drug manufactured and marketed by Ono Pharmaceutical Industries, Ltd. and Bristol-Myers Squibb Co.

The research will be funded by a scholarship donation from the Department of Esophago-Gastroenterological Surgery to which the principal investigator belongs. The scholarship donation does not include funds from Ono Pharmaceutical Industries, Ltd.

**23.2 Involvement in research by companies that manufacture and sell test drugs**

The companies that manufacture and sell the test drugs to be evaluated in this study will not be involved in the research.

**23.3 Involvement of companies that manufacture and sell test drugs and individual researchers**

**23.3.1 Conflicts of interest requiring disclosure**

This study will disclose whether or not there are any persons engaged in the research who have a relationship with the manufacturer or distributor of the drug or its special related parties involved in the research as described in 1) to 6) below.

1)　Donations totaling more than 2 million yen per year are received from target drug pharmaceutical companies.

2)　Total amount of donations provided by target drug pharmaceutical companies and belonging to endowed chairs provided by manufacturers and distributors of pharmaceuticals and other products.

3)　Receives personal benefits totaling 1,000,000 yen or more per year from the covered drug manufacturer to the declarant or spouse and first-degree relatives who share the same livelihood as the declarant or the declarant's spouse or first-degree relative.

4)　He is a member of the board of directors of a target drug pharmaceutical company.

5)　The person reporting the conflict of interest owns stock in or invests in the target pharmaceutical company.

6)　Involvement of the subject drug pharmaceutical company or other entities with respect to other conflict of interest filers.

**23.3.2 Disclosure of conflicts of interest**

There are no conflicts of interest in this clinical research that should be disclosed.

**23.4 Management of conflicts of interest**

The Principal Investigator shall prepare a conflict of interest management plan based on the Conflict of Interest Management Standards and shall manage conflicts of interest in accordance with these standards. The Principal Investigator shall obtain opinions on the conflict of Interest Management Standards and Conflict of Interest Management Plan at the Accredited Clinical Research Review Committee and maintain fairness with respect to the interests in this clinical research.

When a new conflict of interest relationship arises, the person reporting the conflict of interest shall prepare a self-report on the researcher's conflict of interest, obtain confirmation from his institution, and receive a report confirming the conflict of interest status. The person reporting a conflict of interest shall submit a conflict of Interest Status Confirmation Report to the principal investigator. The principal investigator shall prepare a conflict of interest management plan and submit it to the principal investigator.

If there are any changes to the conflict of interest management plan, the Principal Investigator shall obtain the opinion of the Accredited Clinical Research Review Committee on the changes to the conflict of interest management plan in accordance with "Changes to Research Protocol, Procedures, or Statistical Analysis Plan.

When researchers participating in this research publish the results of their research, in addition to the management under this section, they shall publish them in accordance with the rules for the management of conflicts of interest established by the paper and the academic societies.

# 24．Attribution of research results and publication of research findings

## 24.1 ttribution of research results

The results and data obtained from this clinical research and intellectual property rights belong to Kobe University Hospital, Department of Esophago-Gastroenterological Surgery and Department of Oncology and Hematology. The specific handling and allocation of the intellectual property will be decided through consultation. Whether the intellectual property of the principal investigator, Taro Oshikiri, belongs to the individual or to the research institution is subject to the agreement of the research institution to which he belongs.

## 24.2 Publication of research results

## 24.2.1 Publication by jRCT

The Principal Investigator will publish an outline of the study and its implementation in the jRCT, and will update the published information in a timely manner after obtaining the opinion of the Accredited Clinical Research Review Committee when making changes to the published information.

Since the primary and secondary endpoints were collected at the same time in this study, the primary endpoint report will not be prepared; only the summary report and its summary will be prepared. Within one year after the end of the period for collecting data on all evaluation items, a summary report and a summary of the summary report shall be prepared, and the opinions of the accredited clinical research review committee shall be heard. Within one month of receiving an opinion from the Clinical Research Review Committee, submit the opinion to the administrator of the implementing medical institution and publish it in the jRCT. In addition, a summary of the summary report (notification of termination) and the research plan, (statistical analysis plan if prepared) will be submitted to the Minister of Health, Labour and Welfare, and these documents will also be made public.

The principal investigator will provide information to the other principal investigators when the above publication is made. Each principal investigator who receives the information will promptly report it to the administrator of the implementing medical institution.

### 24.2.2 Publication in publications and academic activities

The results of this clinical study will be presented at a conference and submitted as an article to an english-language professional journal. Presentations at conferences in Japan shall also be made as necessary. The first author of the main publication of the research results shall be the Research Office, followed by the Principal Investigator and the Sub-Principal Investigator, in that order. In accordance with the restrictions imposed by the article submission rules, the principal investigators from the implementing medical institutions will be selected as co-authors in the order of the number of enrollments. The final author shall be Yoshihiro Kakeji.

All co-authors must have reviewed the content of the paper prior to submission and agree on the content of the presentation. If there is no agreement on the content after discussion, the principal investigator may choose not to include the investigator as a co-author. Since there may be multiple presentations at the conference, presentations will be made on a rotating basis by the Research Office, the Principal Investigator, and the Principal Investigators or those designated by the Principal Investigators at the implementing medical institutions with the highest enrollment. The presenter will be determined with the approval of the principal investigator. However, the Research Office shall be responsible for the preparation and content of the presentation at the conference.

## 24.3 Data sharing

In this clinical study, data sharing will be conducted after the study is completed. The research protocol and statistical analysis plan will be published in jRCT. Provide the collected data in a non-personally identifiable form in the event that a researcher who has demonstrated an appropriate methodological research plan after publication of the results paper makes an inquiry to the authors of the paper. The term of provision is five years from three months after the publication of the paper.

# 25．Research organization

## 25.1 Research institution

**1. Principal investigator and principal investigator**

**＜Principal investigator＞**

Taro Oshikiri　 Division of Gastrointestinal Surgery, Department of Surgery, Kobe University

**＜Institution/principal investigator＞**

| **Medical institution** | **Affiliation** | **Responsible physician** |
| --- | --- | --- |
| Kobe University Hospital | Division of Gastrointestinal Surgery,　Department of Surgery | Taro Oshikiri |
| Hyogo Cancer Center | Division of Gastrointestinal Surgery | Dai Otsubo |
| Kakogawa Central City Hospital | Division of Gastrointestinal Surgery | Toru Nishimura |
| Saiseikai Nakatsu Hospital | Division of Gastrointestinal Surgery | Masashi Yamamoto |
| Kitaharima Medical Center | Division of Gastrointestinal Surgery | Tetsu Nakamura |
| Awaji Medical Center | Division of Gastrointestinal Surgery | Akio Nakagawa |
| Harima-Himeji General Medical Center | Division of Gastrointestinal Surgery | Takashi Yasuda |
| Meiwa Hospital | Division of Gastrointestinal Surgery | Yoshihiko Nakamoto |
| Konan Medical Center | Division of Gastrointestinal Surgery | Gosuke Takiguchi |

**2. Persons engaged in clinical research other than the principal investigator**

| **Job** | **Medical institution** | **Name** |
| --- | --- | --- |
| Data management | Kobe University Hospital | Hironobu Goto |
| Statistical analysis | Kobe University Hospital | Yohei Funakoshi |
| Monitoring | Kobe University Hospital | Hiroshi Hasegawa |
| Audit | Not applicable |  |
| Research and development planning support | Not applicable |  |
| Coordination and management practices | Not applicable |  |
| Persons overseeing the research other than the principal Investigator | Kobe University Hospital | Yoshihiro Kakeji |

## 25.2 How to respond to consultations with research subjects and related parties

**1) Contact information**

Taro Oshikiri

Division of Gastrointestinal Surgery, Department of Surgery, Kobe University

7-5-2, Kusunoki-cho, Chuo-ku, Kobe, Hyogo, 650-0017, Japan

Reception hours：8：30 ～ 17：15（Excluding Saturdays, Sundays and holidays）

Kobe University hospital, Patient Consultation

Outpatient Care Building, 1st floor, General consultation service

Outpatient Care Bldg. 3rd floor, Center for clinical research promotion, Patient consultation

7-5-2, Kusunoki-cho, Chuo-ku, Kobe, Hyogo, 650-0017, Japan

Reception hours：8：30 ～ 17：00（Excluding Saturdays, Sundays and holidays）

**2) Contact information for medical treatment and consultation**

　　　　　Principal investigators at each insutitution

## 25.3 Details of outsourced operations and outsourcing partners

**＜Outsourcing agency＞**

　【Immunostaining institution】

LSI Medience Inc.

136-3, Tamatsu-cho, Kobe, Hyogo, 651-2122, Japan

# 26．Bibliography and References

1. Sobin L, Gospodarowicz M, Wittekind C. TNM Classification of Malignant Tumours, Seventh edition. 2010.
2. Ando N, Kato H, Igaki H, Shinoda M, Ozawa S, Shimizu H, et al. A randomized trial comparing postoperative adjuvant chemotherapy with cisplatin and 5-fluorouracil versus preoperative chemotherapy for localized advanced squamous cell carcinoma of the thoracic esophagus (JCOG9907). Ann Surg Oncol. 2012;19(1):68-74.
3. Kelly RJ, Ajani JA, Kuzdzal J, Zander T, Van Cutsem E, Piessen G, et al. Adjuvant Nivolumab in resected Esophageal or Gastroesophageal Junction Cancer. N Engl J Med. 2021;384(13):1191-1203.

# 27. Revision history

| Version number | Date of creation and revision | Reason for revision and contents |
| --- | --- | --- |
| Version 1.0 | July 29, 2022 | Newly enacted |
| Version 2.0 | October 10, 2022 | Revision due to items pointed out by the committee, and to change the inspection subcontractor |
| Version 2.1 | December 22, 2022 | To add items to observations, tests, evaluation items, and patient background |
