# Supplementary material for: Protocol for a phase II study to evaluate the efficacy and safety of nivolumab as a postoperative adjuvant therapy for patients with esophageal cancer treated with preoperative docetaxel, cisplatin plus 5-fluorouracil treatment (PENTAGON trial)
Source: PLoS One. 2024 Apr 18;19(4):e0299742. doi: 10.1371/journal.pone.0299742 (PMC11025784; doi:10.1371/journal.pone.0299742)
Supplement: S1 Fig — (DOC) [file pone.0299742.s003.doc]

|  | **STUDY PERIOD** | | | | | | | |
| --- | --- | --- | --- | --- | --- | --- | --- | --- |
|  | **Enrolment** | **Allocation** | **Post-allocation** | | | | | **Close-out** |
| **TIMEPOINT**** | ***-28 days*** | **0** | ***14***  ***days*** | ***3 months*** | ***12 months*** |  | ***60 months*** | ***60 months*** |
| **ENROLMENT:** |  |  |  |  |  |  |  |  |
| **Eligibility screen** | X |  |  |  |  |  |  |  |
| **Informed consent** | X |  |  |  |  |  |  |  |
| **Allocation** |  | X |  |  |  |  |  |  |
| **INTERVENTIONS:** |  |  |  |  |  |  |  |  |
| ***Nivolumab***  ***administration*** |  |  |  |  |  |  |  |  |
| **ASSESSMENTS:** |  |  |  |  |  |  |  |  |
| ***Symptoms*** | X | X |  |  |  |  |  | X |
| ***Adverse event*** |  |  |  |  |  |  |  | X |
| ***Blood examination*** | X |  |  |  |  |  |  | X |
| ***Computed tomograpy*** | X |  |  |  |  |  |  | X |
